# A novel evaluation of endothelial dysfunction ex vivo: “Teaching an Old Drug a New Trick”

**DOI:** 10.14814/phy2.15120

**Published:** 2021-11-10

**Authors:** Lexiao Jin, Daniel J. Conklin

**Affiliations:** ^1^ American Heart Association‐Tobacco Regulation and Addiction Center University of Louisville Louisville Kentucky USA; ^2^ Christina Lee Brown Envirome Institute University of Louisville Louisville Kentucky USA; ^3^ Superfund Research Center University of Louisville Louisville Kentucky USA; ^4^ Diabetes and Obesity Center University of Louisville Louisville Kentucky USA; ^5^ Division of Environmental Medicine Department of Medicine University of Louisville Louisville Kentucky USA

**Keywords:** aldehydes, cardiovascular disease, endothelium, eNOS, L‐NAME

## Abstract

Cardiovascular disease (CVD) is the leading cause of morbidity and mortality worldwide. Many CVDs begin with endothelium dysfunction (ED), including hypertension, thrombosis, and atherosclerosis. Our assay evaluated ED in isolated murine aorta by quantifying phenylephrine‐induced contractions (PE) in the presence of L‐NAME, which blocked acetylcholine‐induced relaxation (ACh %; >99%). The “L‐NAME PE Contraction Ratio” (PECR) was defined as: “PE Tension post‐L‐NAME” divided by “PE Tension pre‐L‐NAME.” We hypothesized that our novel PE Contraction Ratio would strongly correlate with alterations in endothelium function. Validation 1: PECR and ACh % values of naïve aortas were strongly and positively correlated (PECR vs. ACh %, *r* ^2^ = 0.91, *n* = 7). Validation 2: Retrospective analyses of published aortic PECR and ACh % data of female mice exposed to filtered air, propylene glycol:vegetable glycerin (PG:VG), formaldehyde (FA), or acetaldehyde (AA) for 4d showed that the PECR in air‐exposed mice (PECR = 1.43 ± 0.05, *n* = 16) correlated positively with the ACh % (*r* ^2^ = 0.40) as seen in naïve aortas. Similarly, PECR values were significantly decreased in aortas with ED yet retained positive regression coefficients with ACh % (PG:VG *r* ^2^ = 0.54; FA *r* ^2^ = 0.55). Unlike other toxicants, inhaled AA significantly increased both PECR and ACh % values yet diminished their correlation (*r* ^2^ = 0.09). Validation 3: To assess species‐specific dependence, we tested PECR in rat aorta, and found PECR correlated with ACh % relaxation albeit less well in this aged and dyslipidemic model. Because the PECR reflects NOS function directly, it is a robust measure of both ED and vascular dysfunction. Therefore, it is a complementary index of existing tests of ED that also provides insight into mechanisms of vascular toxicity.

## Graphical abstract

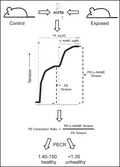

**New & Noteworthy**

Endothelial dysfunction (ED) is *sine qua non* of atherosclerosis and other cardiovascular diseases in humans, yet the mechanisms that contribute to ED are still being investigated. Our study demonstrates a novel methodological twist using an old drug (L‐NAME) to screen for ED in aorta ex vivo.

## 1 INTRODUCTION

Cardiovascular disease (CVD) is the leading cause of morbidity and mortality worldwide (Benjamin et al., 2019; World Health Organization, 2018). Notably, many chronic CVDs are a result of endothelial dysfunction (ED)—vascular injury that increases the risk of hypertension, platelet activation and thrombosis, and atherosclerosis (Conklin et al., 2010; DeJarnett et al., 2014; Sithu et al., 2010; Srivastava et al., 2011; Wheat et al., 2011). The endothelium is a single layer of squamous cells that line the interior surface of 60,000 miles of blood vessels, and thus, it is a major player in cardiovascular homeostasis, and its injury fosters development of CVD. Thus, reliable techniques to measure endothelium dysfunction have emerged over the last 50 years. One approach to evaluate ED is to measure biomarkers in the blood, such as the levels of soluble adhesion molecules, microparticles, endothelial progenitor cells, etc. (Leite et al., 2020; Lynch et al., 2020; O'Toole et al., 2010). A more direct method is to measure the changes in conduit artery diameter by ultrasound following induction of flow‐mediated dilation (FMD) in humans (Celermajer, 1985; Celermajer et al., 1996) and animals in vivo (Heiss et al., 2008). A similar approach is used for the reactive hyperemic flow response in fingertip microcirculation (EndoPAT™; VENDYS™; Bard et al., 2010). Multiple mechanisms likely induce altered endothelium‐dependent responses in FMD, and thus, it is necessary also to test for the effects of an NO donor as a control for altered vascular smooth muscle responsiveness before one can conclude there is ED (Celermajer et al., 1996). Thus, there is an integral dependence of these measurements of endothelium function on nitric oxide (NO), which provides the rationale for our current investigation.

To study nitric oxide synthase (NOS) in vascular control, two drugs became available in the early 1990s: N^ω^‐Nitro monomethyl L‐arginine acetate (L‐NMMA) and N^ω^‐Nitro L‐arginine methyl ester hydrochloride (L‐NAME), and these are used extensively in numerous settings (Alemayehu et al., 1994; Leblais et al., 2008). The latter drug, L‐NAME (aka the “old drug” in the title), perhaps due to its slightly better solubility and availability of a pharmacological control compound (D‐NAME), has remained in popular use since 1990. A recent PubMed search yields over 17,000 hits in the last 30 years starting with just 10 hits in 1990, peaking with nearly 900 hits in the early to mid‐2000s, and hits remain relatively high with >400 in 2020. As L‐NAME inhibits all 3 NOS isoforms, it remains a useful and practical tool for investigating overall NOS influence of vascular function especially in isolated aorta (Jin, Jagatheesan, et al., 2019; Jin et al., 2020; Jin, Lorkiewicz, et al., 2019; Rees et al., 1989).

Herein we asked whether L‐NAME could be more useful in evaluation of vascular dysfunction in naïve aorta, in aorta with intact perivascular adipose tissue (PVAT), and in aorta isolated following in vivo exposures to inhaled toxicants and ingested nicotine, and whether L‐NAME in measurement of ED could be validated in a variety of settings and in more than one species. The aorta is unique in that it relies heavily (if not exclusively) on NOS (eNOS and nNOS) for its endothelium‐dependent relaxation to acetylcholine (ACh; Capettini et al., 2008). Quantifying the ACh‐induced relaxation ex vivo is a classical approach to assess ED in aorta, and likewise, it is common to use L‐NAME pretreatment to inhibit ACh‐induced relaxation, which it does typically >99%. This is not true of other blood vessels that rely more heavily on other endothelium‐derived relaxing factors (EDRFs; e.g., prostacyclin) or endothelium‐derived hyperpolarizing factors (EDHFs) such as H_2_O_2_, K^+^, or EETs (Busse et al., 2002; Chen et al., 1988). For example, ACh‐induced relaxation in isolated murine superior mesenteric artery is inhibited only 15% by L‐NAME indicating the robust release of other EDRF/EDHF in response to ACh (Jin, Jagatheesan, et al., 2019). Thus, our investigation hinges on two important assumptions: (1) aorta is exclusively dependent on NOS for EDRF release following ACh stimulation; and, (2) aortic contractility itself is a robust and integrated measure of overall vascular function. Based on these conditions, we developed a new methodology to assess ED in the isolated aorta. We hypothesized that our novel approach (termed the Phenylephrine Contraction Ratio; PECR) would strongly correlate with alterations in endothelium function. As predicted, our new approach using an “old drug” confirms a traditional measure of ED (i.e., ACh‐induced relaxation in aorta), yet also extends assessment of vascular dysfunction by revealing alterations in aortic contractility that are likewise L‐NAME dependent. By “teaching an old drug (L‐NAME) a new trick,” we aim to augment the productivity and insight of experimenters who evaluate aortic function ex vivo.

## 2 MATERIALS AND METHODS

### 2.1 Materials

Reagent‐grade chemicals were purchased from Sigma‐Aldrich (St. Louis, MO) unless otherwise stated. *Physiological salt solutions* (*PSS*). PSS for aorta was (in mM): NaCl, 118; KCl, 4.7; CaCl_2_, 2.5; KH_2_PO_4_, 1.2; MgSO_4_, 1.2; NaHCO_3_, 25; and, glucose, 5.5; pH 7.4. High K^+^ PSS (100 mM) was same as PSS except for substitution of equimolar K^+^ for Na^+^.

### 2.2 Rodents and exposures

#### 2.2.1 Rodents

Male and female C57BL/6J (12–20 weeks old; wild type, WT) mice and 1‐year old male LDLR‐knockout (LDLR‐KO) and wildtype (WT) counterpart rats were obtained from The Jackson Laboratory (Bar Harbor, ME) or from in‐house breeding colonies, respectively. All animals were treated according to the *Guiding Principles for the Care and Use of Animals in Research and Teaching* as adopted by the American Physiological Society, and all protocols were approved by the University of Louisville Institutional Animal Care and Use Committee. Before and during exposures, rodents were housed under pathogen‐free conditions, controlled temperatures, and a 12:12 h light:dark cycle. Rodents were maintained on a standard chow diet (Rodent Diet 5010, 4.5% fat by weight, LabDiet).

#### 2.2.2 E‐cigarette aerosol and gas exposures

All whole‐body exposures for 4 consecutive days were done between 7 a.m. and 2 p.m. in the absence of food or water as described (Jin et al., 2021).

#### 2.2.3 CAP (concentrated ambient PM_2.5_) exposures

All whole‐body exposures were done for 9 days to HEPA‐filtered air or CAP between 7 a.m. and 2 p.m. in the absence of food or water as described (Haberzettl et al., 2012; O'Toole et al., 2010).

#### 2.2.4 Nicotine exposure

Fresh nicotine bitartrate was prepared in water (100 µg/ml; Malovichko et al., 2019) weekly and provided to rats ad libitum for 52 weeks.

#### 2.2.5 Euthanization

Immediately following the final exposure, rodents were euthanized with sodium pentobarbital (≈150 mg/kg, i.p.), ventral thoracotomy, and exsanguination via right ventricle cardiac puncture for blood collection in EDTA‐coated (0.2 M) syringes. Isolated thoracic aortas were used fresh for functional assays described below.

### 2.3 Evaluation of aortic function

Both naïve male mice and male and female mice exposed to either toxicants or filtered air were euthanized, and the mid‐thoracic aorta was isolated and placed into cold (4℃) physiological salt solution (PSS). Thoracic aorta rings (2–4 mm length) were carefully cleaned (without and with PVAT intact) and prepared for isometric myography as described (Lynch et al., 2020). Rat aortas were isolated, cleaned and treated similarly as for murine aorta.

#### 2.3.1 Aorta isometric myography and the phenylephrine (PE) contraction ratio (PECR)

**FIGURE 1:**
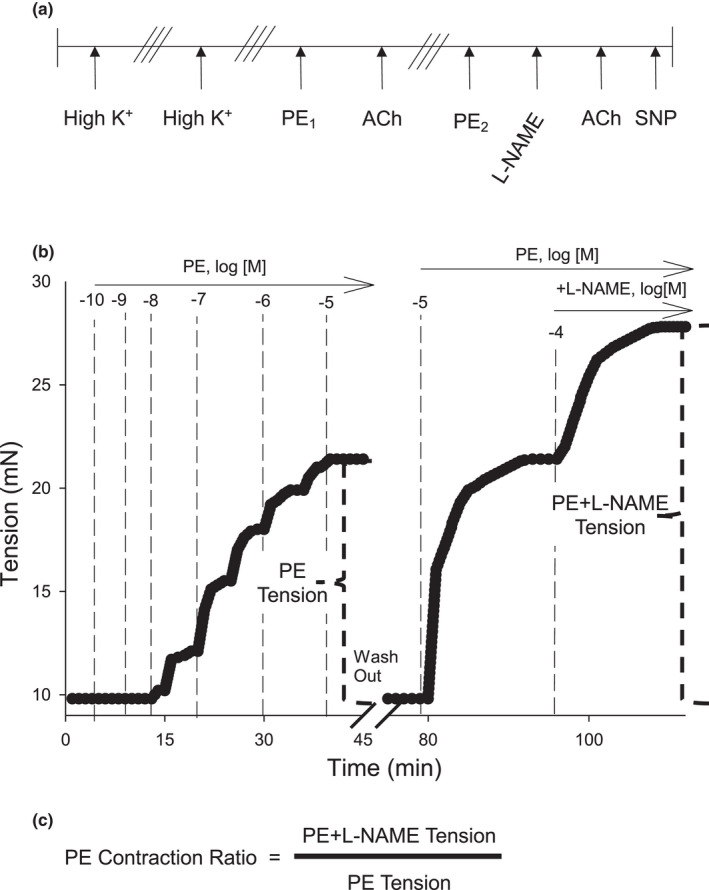
Protocol for development of the Phenylephrine Contraction Ratio (PECR) assay in isolated murine aorta. (a) All aortas were stimulated with High K^+^ followed by washout (i.e. 3 bath exchanges of fresh PSS; ///), and restimulated with High K^+^ followed by washout. Uncontracted vessels were then exposed to cumulative concentrations of PE (0.1 nM–10 µM) before addition of cumulative concentrations of acetylcholine (ACh; 0.1 nM–10 µM). Following washout, aortas were pre‐contracted with PE (10 µM) until a tension plateau occurred, after which, L‐NAME (100 µM) was added to the bath. After a second plateau in tension, ACh (10 µM) was added to confirm NOS inhibition. Finally, aortas were relaxed with cumulative concentrations of sodium nitroprusside (SNP; 0.01 nM–100 µM). (b) Representative traces of contractions induced by PE alone or PE+L‐NAME. Both cumulative concentrations of PE (up to 10 µM) and PE alone at 10 µM produced similar levels of tension. L‐NAME addition significantly increased tension in a time dependent manner. (c) The PECR was calculated as the PE+L‐NAME tension divided by the PE alone tension

Mid‐thoracic (located 0.8–1 cm from aortic arch apex) aortic segments were cut, cleaned of perivascular adipose tissue (or PVAT left intact), and mounted in one of two systems: (1) horizontal pin (DMT, Ann Arbor, MI); or, (2) vertical wire system. In the horizontal pin system, a short, aortic segment (≈2 mm) was mounted on stainless steel pins in a 5‐ml heated (37℃) organ bath bubbled with 95% O_2_:5% CO_2_. One pin was connected to an isometric strain gauge transducer, and the other was attached to a micrometer. Transducer signals were fed directly into a PC with LabChart software (v. 8; ADI). In the vertical wire system, an aortic segment (3–4 mm) was placed on wire hooks (100 µm dia. tungsten wire) with a hook connected to an isometric strain gauge transducer (Kent Scientific, Litchfield, CT) and the other attached to a fixed support glass rod in a 15‐ml heated (37℃) organ bath bubbled with 95% O_2_:5% CO_2_. Transducer signals were fed into an 8‐channel PowerLab A/D converter and recorded on a PC using LabChart software (v. 4.3.2; ADI). Prior to each use, each system was calibrated using standardized weights. In both systems, after 10 min without tension, aortic rings were equilibrated to ≈9.81 mN (≈1 g) loading tension over 30 min before agonist stimulation. All segments were stimulated with High K^+^ to test for viability, washed three times with PSS over 30 min, re‐equilibrated to ≈1 g resting tension, and then stimulated again with High K^+^, followed by three bath changes and re‐equilibration to resting tension (Figure 1a; Jin et al., 2018).

To calculate the PE Contraction Ratio (PECR), aortas were contracted with PE (0.1 nM–10 µM) until a plateau in tension was reached and then relaxed with acetylcholine (ACh; 0.1 nM–10 µM). After three bath changes, each aortic segment was re‐equilibrated to resting tension. Aortas again were contracted with PE (10 µM) and when a plateau in tension was reached, L‐NAME (100 µM) was added and a second plateau in tension was observed. Upon tension plateau, ACh (10 µM) was added. Following ACh, sodium nitroprusside (SNP; 0.01 nM–10 µM; NO donor) was added (Figure 1a). To quantify alterations in endothelial nitric oxide synthase (eNOS) function, the effect of L‐NAME on PE‐induced tension was calculated as a “PE Contraction Ratio” wherein the PE+L‐NAME Tension was divided by the PE alone Tension (Figure 1b,c).

We also tested whether intentional injury to the naïve aortic endothelium affected the PECR value. ED was induced in an aortic segment by room air perfusion (aquarium pump) for 5 min via a cannula inserted into one end of the aorta. Use of the aortic segment then followed the protocol as described above except that the PE tension was divided by High K^+^ tension (surrogate marker of vascular smooth muscle response) because L‐NAME had no effect and a PECR value could not be calculated under these intentional ED conditions.

### 2.4 Statistics

Data are presented as mean ± standard error of mean (*SE*). For two group comparisons, Rank Sum tests with Bonferroni's post‐test or paired or unpaired *t*‐tests as appropriate were applied. For multiple group comparisons, Kruskal–Wallis ANOVA on ranks with Dunn's post‐test were used (SigmaPlot, ver. 12.5; Systat Software, Inc.). Statistical significance was set at *p* < 0.05.

## 3 RESULTS

To confirm that our new approach complements existing measures of endothelial dysfunction (ED) ex vivo, we performed a series of validation experiments. These approaches included using naïve aorta, retrospective analyses of studies that demonstrated toxicant‐induced ED, and the use of two rodent species to test that our method was sensitive enough to detect induced vascular injury. Collectively, these validation studies provide evidence to support the veracity of our new methodological approach using L‐NAME, an old drug.

### 3.1 Validation Study #1

**FIGURE 2:**
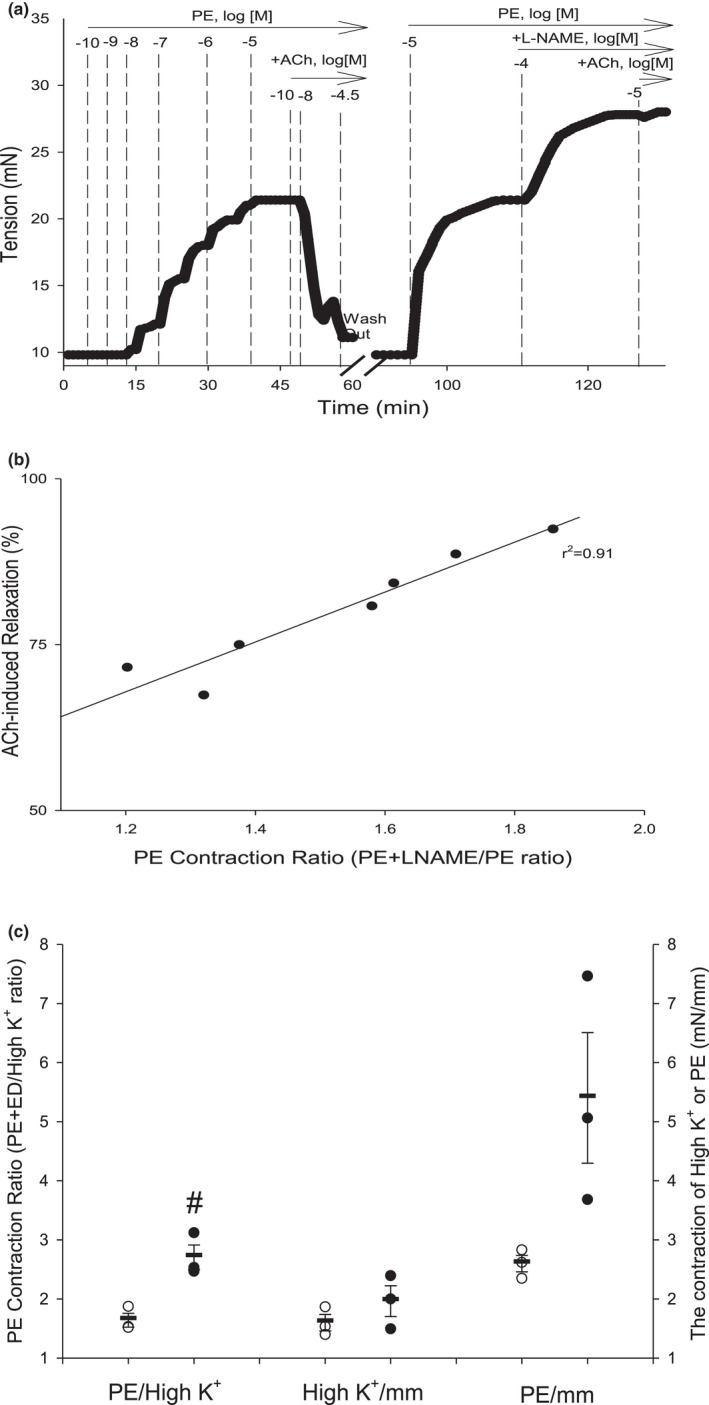
Relationship between Phenylephrine (PE) Contraction Ratio and ACh‐induced endothelium‐dependent relaxation in aortas of naïve mice. (a) Representative myograph trace of isometric tension of a naïve aortic segment contracted by PE and relaxed by acetylcholine (ACh % relaxation). After 3× washout over 30 min, the aorta was contracted again with PE followed by addition of L‐NAME. After a plateau in tension, ACh was added to confirm L‐NAME‐induced inhibition of endothelium‐dependent relaxation (EDRF; NO). The PE Contraction Ratio (PECR) was calculated as the total tension of PE+L‐NAME divided by the total tension generated by PE alone (1st addition; see Figure 1a). (b) The values of PECR (*x*‐axis) were regressed against the values of % ACh relaxation (*y*‐axis) with a regression coefficient of *r* ^2^ = 0.91. (c) Air perfusion‐induced endothelial dysfunction (ED) in aorta led to significantly increased PE‐stimulated contractility reflected by an increased PE+ED/HI K^+^ ratio and by an increased PE efficacy (mN/mm) without altering High K^+^ efficacy (mN/mm)

Part (A) PECR and ACh % relaxation values in naïve aortas were strongly and positively correlated (PECR vs. ACh %, *r* ^2^ = 0.91, *n* = 7; Figure 2a,b). Following L‐NAME and tension plateau, addition of ACh produced minimal relaxation (<2%) indicating nearly 100% inhibition of NOS. These data demonstrate the strong relationship between PECR (a wholly contraction‐based measurement) and the functional status of the endothelium. Also, one can observe real time the effect of NOS inhibition on tension development and that the plateau in tension corresponded with nearly 100% inhibition of ACh‐induced relaxation. As these were naïve aortas of healthy C57BL/6J male mice, the amount of injury to the endothelium (i.e., minimal injury due to careful handling and cleaning) was solely a consequence of preparation and not due to some other extrinsic factor such as was tested for more directly in other validation experiments (see below). Part B) Thus, to control for extrinsic factors, we also tested whether ‘intentional induction’ of ED (i.e., air perfusion for 5 min) altered the PECR alongside impairment of the ACh‐induced relaxation. As expected, air perfusion renders the endothelium nearly unresponsive to ACh, however, impairing the endothelium inactivates NOS, and thus, it makes measurement of the L‐NAME‐dependent response moot, which nevertheless also is a form of validation. To address this confounding limitation, though, we used a surrogate contraction‐based ratio (i.e., PE/HIK^+^ ratio) to evaluate whether intentional ED by air perfusion promotes PE‐stimulated contraction. As HIK^+^ depolarizes all smooth muscle, it was unaffected by air perfusion, whereas PE contraction was increased significantly as reflected by increased PE efficacy (mN/mm) and the PE/HIK^+^ ratio (by 65 ± 21%; Figure 2c). Thus, as these data mirror the effect of L‐NAME treatment in a normal healthy aorta (i.e., increased PECR by approximately 50%), we show the PECR tracks the full continuum of ED‐induced changes in PE contractility in aorta (as hypothesized).

### 3.2 Validation Study #2

ED is a common and sensitive ex vivo biomarker of inhaled toxicant exposures including cigarette smoke (Conklin et al., 2009). Female mice were exposed to e‐cigarette fluid‐derived toxicants for 4 consecutive days, and then aortas were isolated and vascular function assessed by isometric myography ex vivo.

**FIGURE 3:**
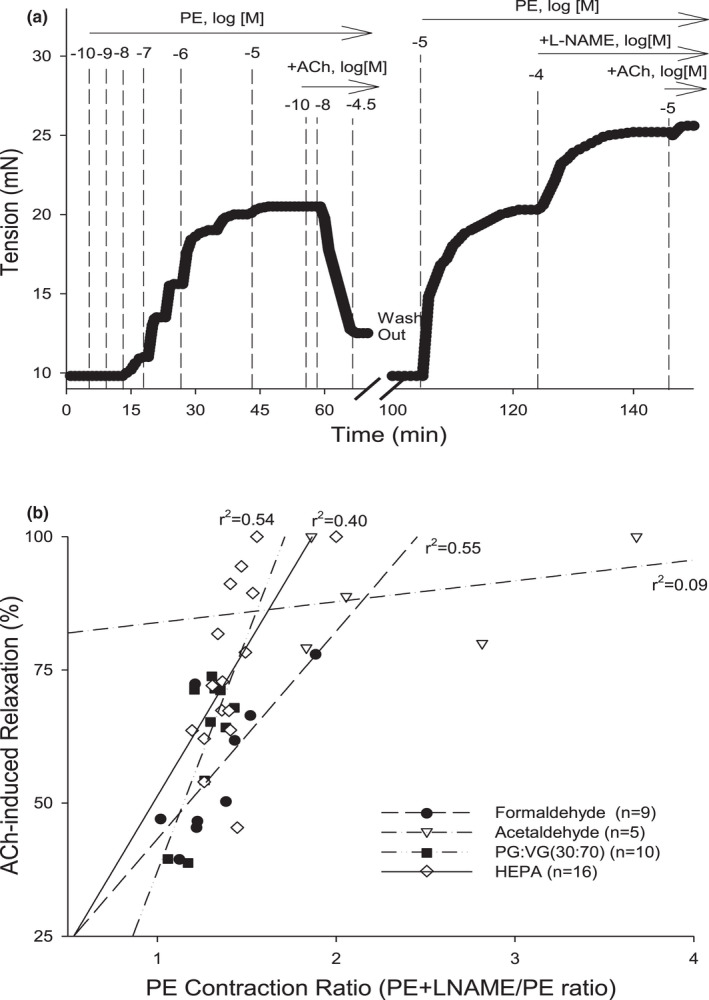
Relationship between Phenylephrine (PE) Contraction Ratio and ACh‐induced endothelium relaxation in aortas of mice exposed to constituents of electronic cigarette aerosols. Retrospective analyses of published PECR and ACh % data of ex vivo aortic function of female mice exposed to filtered air control, propylene glycol: vegetable glycerin (PG:VG), formaldehyde (FA), or acetaldehyde (AA) for 4 days, 6 h/day. (a) Representative myograph trace of isometric tension of an aortic segment contracted by PE and relaxed by acetylcholine (ACh % relaxation). After 3× washout over 30 min, the aorta was contracted again with PE followed by addition of L‐NAME. After a plateau in tension, ACh was added to confirm L‐NAME‐induced inhibition of endothelium‐dependent relaxation (EDRF; NO). The PE Contraction Ratio (PECR) was calculated as the total tension of PE+L‐NAME divided by the total tension generated by PE alone (1st addition; see Figure 1a). (b) The values of PECR (*x*‐axis) were regressed against the values of ACh % relaxation (*y*‐axis) for filtered air control PG:VG, FA, and AA with regression coefficients of *r* ^2^ = 0.40, *r* ^2^ = 0.54, *r* ^2^ = 0.55, and, *r* ^2^ = 0.09, respectively

Part (A) Retrospective analyses of our published PECR and ACh % data of female mice exposed to HEPA‐filtered air, PG:VG, formaldehyde (FA), or acetaldehyde (AA) for 4 days (6 h/day) showed that the PECR in air‐exposed mice (PECR = 1.43 ± 0.05, *n* = 16) correlated positively with the ACh % (*r* ^2^ = 0.40) as it did in naïve aortas (Figure 3a,b; Table 1; Jin et al., 2021). Similarly, PECR values were significantly decreased in aortas with ED, yet retained positive regression coefficients (PG:VG, *r* ^2^ = 0.54; FA, *r* ^2^ = 0.55; Figure 3b). Unlike the other toxicants, AA exposure significantly increased both PECR and ACh % relaxation values, yet greatly diminished the regression between the two parameters indicating a distorted relationship between endothelium‐derived and contractile functions (*r* ^2^ = 0.09; Figure 3b).

**TABLE 1:** Phenylephrine contraction ratio (PECR) and ACh % relaxation values and regression coefficients across all “Validation Studies”

Validation Study	PECR	ACh % relaxation	*r* ^2^ (PECR vs. ACh %)	*n*
1. Naïve	1.52 ± 0.09	−80.0 ± 3.5	0.91	7
2A. Filtered air	1.43 ± 0.05	−75.2 ± 4.1	0.40	16
2A. PG:VG	1.28 ± 0.03*	−61.8 ± 4.2*	0.54	10
2A. FA	1.34 ± 0.09**	−56.3 ± 4.5*	0.55	9
2A. AA	2.45 ± 0.36*	−89.6 ± 4.6*	0.09	5
2B. HEPA (clean)	1.59 ± 0.08	−69.9 ± 3.3	0.26	5
2B. HEPA+PVAT	1.92 ± 0.16**	−86.5 ± 3.6	0.44	5
2B. CAP (clean)	1.57 ± 0.10**	−67.9 ± 4.5	0.02	5
2B. CAP+PVAT	1.91 ± 0.14*	−85.3 ± 6.7	0.70	5
3. WT rat	1.48 ± 0.09	−49.5 ± 5.9	0.28	5
3. WT rat+nicotine	1.43 ± 0.10	−61.8 ± 5.0	0.13	6
3. LDLR‐KO rat	1.28 ± 0.05	−50.8 ± 5.3	0.06	6
3. LDLR‐KO rat+nicotine	1.22 ± 0.03**	−51.2 ± 8.3	0.21	6

Note

Values = mean ± SEM.

Abbreviations: AA, acetaldehyde; ACh, acetylcholine; CAP, concentrated ambient particulate matter; FA, formaldehyde; HEPA, high efficiency particulate air filter; LDLR‐KO, low‐density lipoprotein receptor‐knockout; PG:VG, propylene glycol:vegetable glycerin; PVAT, perivascular adipose tissue; WT, wildtype.

* *p* < 0.05 versus matched control group.

** 0.05 < *p *< 0.10 versus matched control group.

**FIGURE 4:**
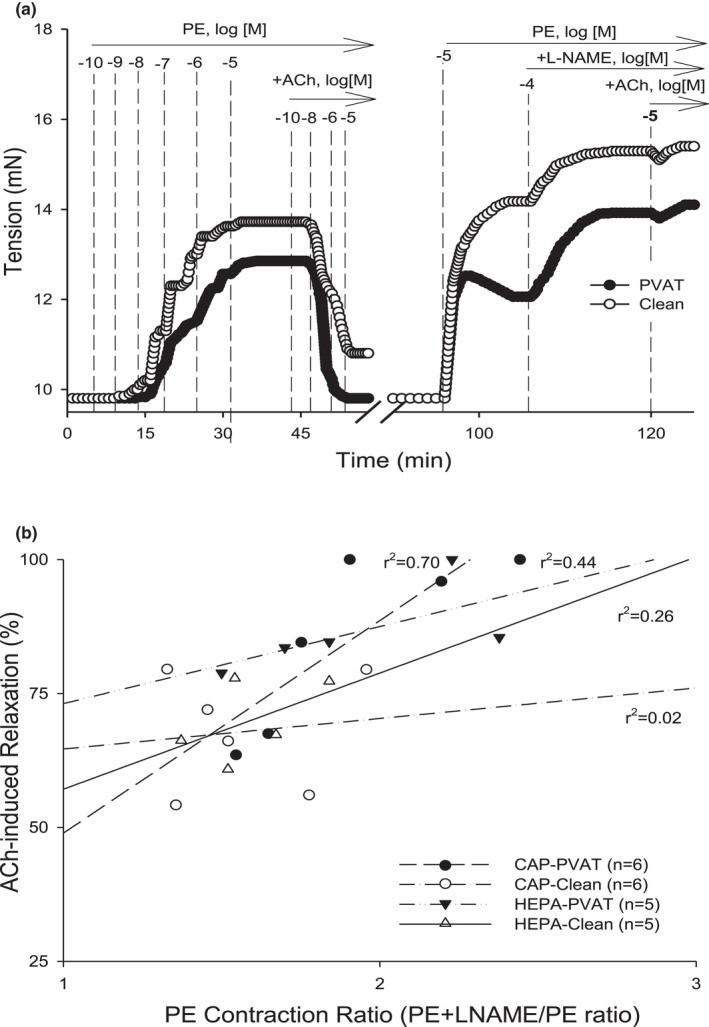
Relationship between Phenylephrine (PE) Contraction Ratio and ACh‐induced endothelium‐dependent relaxation in aortas of mice exposed to concentrated ambient particulate matter (CAP). Retrospective analyses of published PECR and ACh % data of ex vivo aortic function (without and with perivascular adipose tissue (PVAT) intact) of male mice exposed to filtered air control or CAP for 9 days, 6 h/day. (a) Representative myograph traces of isometric tension of aortic segments without (clean) and with PVAT intact contracted by PE and relaxed by acetylcholine (ACh % relaxation). After 3× washout over 30 min, the aortas were contracted again with PE followed by addition of L‐NAME. After a plateau in tension, ACh was added to confirm L‐NAME‐induced inhibition of endothelium‐dependent relaxation (EDRF; NO). The PE Contraction Ratio (PECR) was calculated as the total tension of PE+L‐NAME divided by the total tension generated by PE alone (1st addition; see Figure 1a). (b) The values of PECR (*x*‐axis) were regressed against the values of % ACh relaxation (*y*‐axis) for filtered air control (HEPA) and CAP (clean and PVAT) with regression coefficients of HEPA‐Clean (*r* ^2^ = 0.26), HEPA‐PVAT (*r* ^2^ = 0.44), CAP‐clean (*r* ^2^ = 0.02), and, CAP‐PVAT (*r* ^2^ = 0.70), respectively

Part (B) In a second retrospective study, we examined the effects of a different exogenous pollutant exposure (concentrated ambient particulate matter, CAP) on aorta function ex vivo, and we also included aorta with perivascular adipose tissue (PVAT) intact to assess how the PECR assay performed in the presence of anticontractile PVAT (Figure 4a). Chronic CAP exposure is known to induce vascular dysfunction, but we used an acute exposure protocol (9‐day) that is known to stimulate early changes in vascular signaling (insulin and VEGF resistance) and inflammation that portend endothelial dysfunction and favor pro‐atherosclerotic changes. Unsurprisingly, both clean and PVAT intact aorta from the control group (filtered air, HEPA) had strong relationships between PECR and % ACh relaxation values with regression coefficients of 0.26 and 0.44, respectively (Figure 4b; Table 1). In fact, PVAT appeared to strengthen the regression coefficient. In the CAP‐exposed groups, the relationship between PECR and % ACh relaxation values in the clean aortas was minimal (*r* ^2^ = 0.02), whereas in aorta with PVAT intact, the correlation was strong at *r* ^2^ = 0.70 indicating that CAP exposure had disturbed the general relationship between vascular contractility and endothelial relaxation—a deleterious change that appeared compensated in the presence of PVAT.

### 3.3 Validation Study #3

**FIGURE 5:**
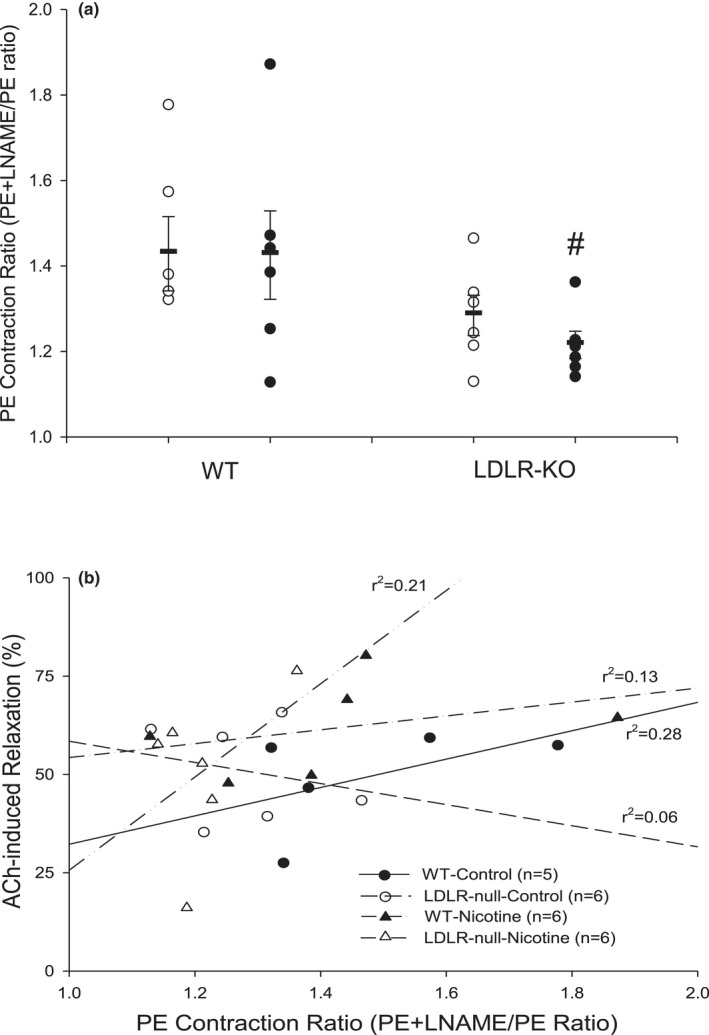
Relationship between Phenylephrine (PE) Contraction Ratio and ACh‐induced endothelium relaxation in aortas of 1‐year‐old male WT and LDLR‐KO rats exposed to water or nicotine in water. (a) Graph summary of the aortic PE Contraction Ratio (PECR) across four groups of rats. (b) The values of PECR (*x*‐axis) were regressed against the values of % ACh relaxation (*y*‐axis) for WT (water control), WT+nicotine, LDLR‐KO and LDLR‐KO+nicotine with regression coefficients of WT (*r* ^2^ = 0.28), WT+nicotine (*r* ^2^ = 0.13), LDLR‐KO (*r* ^2^ = 0.06), and, LDLR‐KO+nicotine (*r* ^2^ = 0.21), respectively. Values are means ± *SE* of 5–6 aortas per group. #, 0.05 < *p *< 0.10 versus WT water control

To address a potential species‐specific dependent effect of our approach, we also tested whether the PECR was associated with ACh % relaxation in isolated thoracic aorta of male LDLR‐KO rats and their WT counterparts. As in naïve murine aortas, the average PECR value in WT rat aortas was approximately 1.5 (Figure 5a; Table 1). Similar to that observed in naïve murine aortas, the PECR and ACh % relaxation values in WT rat aortas were positively correlated (PECR vs. ACh %, *r* ^2^ = 0.28, *n* = 5; Figure 5b). The PECR value was unaffected by nicotine treatment in WT rats, but the average PECR value appeared lower, in general, in LDLR‐KO rats without and with nicotine treatment (Figure 5a). Furthermore, the association between PECR and ACh % relaxation values was likewise diminished in LDLR‐KO aortas and, surprisingly, somewhat restored in aortas of LDLR‐KO rats with nicotine treatment (Figure 5b).

## 4 DISCUSSION

Our new approach that derives a PE Contraction Ratio (ratio of PE‐induced tension post‐L‐NAME:pre‐L‐NAME) has several important benefits for evaluation of vascular function ex vivo. (1) We show PECR closely tracks even subtle injury to the endothelium and thereby provides both confirmation of classical ED measurements, for example, ACh‐induced relaxation, as well as being an independent measure that stands alone. (2) It has a robust baseline ratio of 1.5, so the ratio can go down (lower = ED) or go up (higher = contractile dysfunction). This separates it from the direct measurement of ACh‐induced relaxation, which in many cases cannot detect an enhanced relaxation apart from that found in a naïve aorta (i.e., robust relaxation). (3) Although classical ED as measured by a reduction in ACh‐induced relaxation is a “gold standard” of ex vivo measures, it requires that one measure aortic sensitivity to a non‐endothelium source of NO to rule out changes in the smooth muscle sensitivity to NO itself (i.e., an NO donor such as SNP). Because L‐NAME inhibits all NOS isoforms, one can assume that changes in the PECR value are NOS‐dependent. (4) The approach does not add any extra work but only requires *that L*‐*NAME be added after PE rather than before it—it is just that simple*. This minor protocol modification makes the ratio possible, and as many investigators are already using L‐NAME in their aortic preparations, this adds no additional effort. (5) Changes in PE‐induced tension upon addition of L‐NAME are real‐time, and thus, kinetic changes in endothelial and smooth muscle cell function are dependent on NOS inhibition as it occurs. This affords obvious opportunities for measurement of other parameters that may be contributing to overall contraction (e.g., Ca_i_^++^, NO formation, H_2_O_2_ formation, superoxide, prostanoids, ET‐1, etc…). Prior work in murine aorta indicates that both eNOS‐derived NO and nNOS‐derived H_2_O_2_ contribute to ACh‐induced relaxation (Capettini et al., 2008). (6) It reveals a functional contribution of PVAT to aortic contractility, which is a novel finding of PECR that allows for more detailed exploration of the role of PVAT‐derived NO in vascular control (Gil‐Ortega et al., 2010). And, (7) the PECR value is not species specific as we show it is applicable both in mouse and in rat aorta.

### 4.1 PECR reveals classic and novel forms of endothelium dysfunction: Role of NOS

The PE Contraction Ratio is a robust measure because the efficacy of L‐NAME inhibition of NOS is supported by near abolition of ACh‐induced relaxation (>99% block) indicating the aorta of exposed mice remains completely dependent on NOS and not another source of EDRF/EDHF as in other blood vessels (Lynch et al., 2020). Thus, these data support that the PE Contraction Ratio reflects a continuum of endothelium function including the deleterious effects of PG:VG and FA exposures on ACh‐induced relaxation. eNOS uncoupling due to oxidation of tetrahydrobiopterin (BH_4_) is a known mechanism of ED due to PM_2.5_ exposure (and other conditions) in rodents (Campen, 2009), and although the mechanism by which PG:VG (or FA) exposure induces ED remains uncertain, the PECR data indicate it is NOS‐dependent (e.g., down‐regulation or inhibition of endothelial NOS isoforms) as in agreement with the acute onset of ED and specific loss of eNOS (or nNOS) bioactivity recorded in recent human and animal e‐cigarette exposure studies (Carnevale et al., 2016; Fetterman et al., 2018; Rao et al., 2020).

In our retrospective analysis, we find that exposure of mice to acetaldehyde (AA) gas significantly increases both the PECR and the ACh‐induced relaxation in isolated aorta, yet there is minimal correlation between the 2 parameters (*r* ^2^ = 0.02). If AA exposure enhances endothelium‐dependent response to ACh via NOS activation (e.g., phosphorylation of Ser^1177^), we expect the PECR to track this effect and that the correlation between PECR and ACh % relaxation values remain high (e.g., *r* ^2^ > 0.50), but the loss of and flattening of the correlation between PECR and ACh % values indicate that PECR increases more than the ACh % relaxation increases, which likely reflects an uncoupling of NOS, and perhaps, more superoxide formation. Whether this speculation is correct remains to be tested, but, nonetheless, the PECR measurement adds insight that would otherwise be absent if only the ACh % relaxation was measured.

There are limitations to our approach that warrant mentioning. The PECR value may be agonist‐dependent as our testing focused solely on PE, an α_1_‐adrenergic receptor agonist, so this will need to be tested. We also did not test the effects of D‐NAME, a pharmacological control for L‐NAME, which may be a minor oversight. More importantly, we did not compare L‐NAME effects on PECR values with effects of any other NOS inhibitor such as L‐NMMA, although we feel that L‐NMMA (or any other L‐arginine analogue) likely will work equally well as L‐NAME (Cotter et al., 2000). There are, of course, quantitative and qualitative differences between these NOS inhibitors that include their specific NOS isoform IC_50_s, L‐NAME is a pro‐drug ester (Moroz et al., 1998; Rees et al., 1990), and that L‐NAME has the chemical capability to release NO from the guanidino nitro group (Liu et al., 2019) although this latter phenomenon likely has minimal effect under our conditions. Also, L‐NAME blocks all 3 isoforms, so we cannot assess the contribution of any specific NOS isoform. Although we embrace this non‐selective quality of L‐NAME as a strength, follow up experiments with selective inhibitors of nNOS (e.g., 7‐nitroindazole sodium salt) and iNOS (e.g., 1400W) can be performed to test their contributions apart from eNOS, especially in some of the toxicological settings explored herein where induction of deleterious pathways (e.g., enzyme uncoupling, free radicals) in the aorta and PVAT may occur (Cardounel et al., 2007; Kopincova et al., 2012; Sun et al., 2008).

### 4.2 Conclusions

Because the PECR reflects NOS function directly, it is a robust tool for evaluating vascular function and predicting ED, and thus, it provides an additional *ex vivo* approach for assessing vascular toxicity of aortas from animals under a variety of conditions including exposures to tobacco product‐derived aerosols and gases. As such, vascular physiologists and toxicologists can benefit by using this “new trick with an old drug.”

## CONFLICTS OF INTEREST

All authors declare no conflicts of interest in this paper. The content is solely the responsibility of the authors and opinions shared in this publication do not necessarily represent the official views of the National Institutes of Health, the Food and Drug Administration, the U.S. Environmental Protection Agency, or the American Heart Association.

## AUTHOR CONTRIBUTIONS

LJ and DJC planned, conducted experiments, and generated, analyzed and interpreted data, and wrote the manuscript. DJC is the guarantor of this work. As such, he had full access to all data and takes responsibility for the integrity and accuracy of the data.

## Data Availability

The datasets used and/or analyzed during the current study are available from the corresponding author upon reasonable request.
